# The Evaluation of Skin Infiltration in Mycosis Fungoides/Sézary Syndrome Using the High-Frequency Ultrasonography

**DOI:** 10.3390/jcm14207143

**Published:** 2025-10-10

**Authors:** Hanna Cisoń, Alina Jankowska-Konsur, Rafał Białynicki-Birula

**Affiliations:** 1 University Centre of General Dermatology and Oncodermatology, Wroclaw Medical University, 50-556 Wroclaw, Poland; alina.jankowska-konsur@umw.edu.pl; 2 Faculty of Medicine, Wroclaw University of Science and Technology, 50-370 Wroclaw, Poland; rafal.bialynicki-birula@pwr.edu.pl

**Keywords:** mycosis fungoides/diagnostic imaging, Sézary syndrome/diagnostic imaging, ultrasonography

## Abstract

**Background/Objectives:** High-frequency ultrasonography (HFUS) has gained increasing attention in dermatology as a non-invasive imaging technique capable of visualizing cutaneous structures with high resolution. In cutaneous T-cell lymphomas (CTCL), including mycosis fungoides (MF)/Sézary syndrome (SS), HFUS may provide an objective method for assessing disease activity and monitoring treatment response. This study aimed to evaluate the clinical utility of HFUS in detecting therapy-induced changes in subepidermal low-echogenic band (SLEB) thickness. **Methods:** We conducted a prospective, single-center study between May 2021 and May 2025. Thirty-three patients with histologically confirmed MF (*n* = 31) or SS (*n* = 2) underwent HFUS at baseline and after 4–8 weeks of treatment. SLEB thickness was measured before (E1) and after early treatment (E2). Patients received systemic agents, phototherapy, or topical regimens. Statistical analysis included mixed-model ANOVA with repeated measures to assess SLEB changes, and post hoc tests were applied to explore the influence of therapy type, age, and gender. **Results:** Among 31 evaluable patients with MF, HFUS revealed a significant reduction in SLEB thickness after treatment (0.90 ± 1.10 mm vs. 0.69 ± 0.89 mm; *F*(1,29) = 8.88, *p* = 0.006, *η*^2^ = 0.23). The type of early therapy (systemic vs. topical) did not significantly affect outcomes (*p* = 0.452). Age emerged as a relevant factor: patients ≥ 66 years exhibited higher baseline SLEB values and a significant reduction post-treatment (*p* < 0.001), whereas no comparable effect was observed in younger patients. Gender did not significantly influence SLEB changes. **Conclusions:** HFUS is a sensitive and clinically applicable imaging tool for monitoring treatment response in MF/SS. Reductions in SLEB thickness were observed across therapeutic modalities and aligned with early clinical improvement. HFUS may serve as a valuable adjunct to standard clinical and histopathological evaluation in the routine management of MF/SS.

## 1. Introduction

Ultrasonography is a well-established imaging modality in various medical fields, appreciated for its non-invasive nature and ability to provide real-time visualization of internal structures. In dermatology, the application of the high-frequency ultrasonography (HFUS) has gained growing interest, particularly for the assessment of the skin and its appendages. Frequencies in the range of 18–22 MHz offer high spatial resolution with penetration allowing for detailed imaging of the skin layers [1]. These ultrasonographic images closely correspond to histological architecture, enabling more accurate evaluation of cutaneous structures [2]. The ability of the HFUS to quantify structural parameters such as skin thickness, echogenicity, and lesion volume may provide clinicians with an objective and reproducible method for assessing disease response. The HFUS may serve as a valuable adjunct in the clinical management of the cutaneous T-cell lymphoma (CTCL) and offers potential advantages in monitoring disease progression, assessing lesion characteristics, and evaluating therapeutic efficacy [3]. CTCL represents a diverse group of non-Hodgkin lymphoid malignancies defined by the clonal proliferation and cutaneous infiltration of neoplastic T lymphocytes. Approximately 75% of primary cutaneous lymphomas originate from T-cell lineages, with nearly two-thirds of these cases classified as either mycosis fungoides (MF)/Sézary syndrome (SS) [4]. The age-adjusted annual incidence of CTCL is 6.4 cases per million individuals. Incidence rates are higher in individuals of African descent compared to those of European descent and greater in males than in females [5,6]. In our study, we focused on two diseases within the spectrum of CTCL, i.e., MF/SS. Diagnosing MF, especially in early patch or plaque stages, is challenging due to mimic benign dermatoses like eczema, psoriasis or adult-onset atopic dermatitis (AD) [7,8]. By contrast, SS represents the leukemic variant of CTCL. It is clinically characterized by the triad of generalized erythroderma, palmoplantar keratoderma, and intense pruritus, accompanied by significant peripheral blood involvement, including the presence of circulating malignant T cells (Sézary cells) can also be easily misdiagnosed with AD [9,10]. As a result, patients frequently face diagnostic delays, with a median time to diagnosis of 3–4 years, though in some cases, it may take several decades [11]. Patients with early-stage mycosis fungoides generally exhibit a favorable prognosis, but with advanced-stage MF, the 5-year survival rate is approximately 40%. Consequently, early and accurate diagnosis, along with prompt initiation of therapy, is critically important for improving clinical outcomes in MF/SS. In HFUS assessment of MF/SS, a characteristic finding is the presence of a markedly thickened subepidermal low-echogenic band (SLEB), which reflects extensive infiltration of atypical lymphocytes within the dermis. This echolucent band, located immediately beneath the epidermal entry echo, serves as a key diagnostic and monitoring feature. A significant decrease in its thickness or complete resolution following therapeutic intervention is considered a reliable marker of clinical improvement and histopathological regression. This pattern of change parallels that observed in certain inflammatory dermatoses, supporting the utility of HFUS in evaluating both disease activity and therapeutic efficacy in cutaneous lymphoproliferative disorders (Table 1) [12,13]. The primary endpoint of our study was the short-term treatment response in MF/SS assessed by HFUS, and the secondary endpoint was the evaluation of HFUS as a non-invasive monitoring tool.

## 2. Materials and Methods

A prospective, single-center study was conducted at the University Centre of General Dermatology and Oncodermatology, in 4 years period from October 2021, to evaluate patients with MF/SS. Patients were consecutively screened for eligibility. Inclusion criteria were: (1) age > 18 years and (2) diagnosis confirmed independently by two senior dermatologists based on clinical and histological examination and (3) the patient provided informed consent to participate in the study. Exclusion criterium were the occurrence of other dermatological diseases besides MF/SS. A total of 36 patients were initially recruited, and after applying the inclusion and exclusion criteria, 33 patients were included in the final study cohort. The cohort comprised 31 patients with MF, and 2 with SS. Finally, exclusion of patients with SS from the statistical analyses was undertaken to avoid limiting the conclusions and to maintain the generalizability of the findings. The study was conducted in accordance with the Declaration of Helsinki and approved by the Ethics Committee of The Bioethics Committee at the Wroclaw Medical University (protocol code KB-851/2021, date of approval 28 October 2021). Written informed consent was obtained from the participants prior to enrollment. Data confidentiality and patient privacy were strictly maintained throughout the study.

### 2.1. High-Frequency Ultrasonography (HFUS)

The high-frequency ultrasonography (HFUS) imaging of the skin was performed using a diagnostic system manufactured by taberna pro medicum GmbH (tpm, Lüneburg, Germany). The imaging system was equipped with the 22.5 MHz linear transducer designed for high-resolution dermatologic assessment. The transducer scanned a tissue area with a width of 100 µm and a longitudinal of 12.8 mm, with a declared maximum penetration depth of 8 mm. The axial (vertical) resolution was 80 µm, and the lateral (horizontal) resolution was 200 µm. Ultrasound data acquisition and image storage were managed using the DUBmicro^®^ software version 2017(tpm, Lüneburg, Germany), which facilitated both A-mode and B-mode evaluations. Echogenicity was assessed by measuring the average amplitude of the reflected signal across a normalized scale comprising 255 discrete grayscale levels. In B-mode imaging, hyperechoic structures were visualized as bright regions, while hypoechoic structures appeared darker, corresponding to lower acoustic reflectivity.

### 2.2. Statistics

The study analyzed changes in a subepidermal low-echogenic band (SLEB), a hypoechoic (low-reflectivity) zone immediately beneath the epidermal entry echo, frequently observed in HFUS of inflammatory and lymphoproliferative dermatoses. In the MF/SS thickness of the SLEB correlate with dermal infiltration with atypical lymphocytes and thus may serve as a non-invasive surrogate marker of disease activity and treatment response. The primary variable was the difference between initial examination (E1) and follow-up HFUS measurements (E2) so there were E1–E2, measure units were millimeters. To check whether the type of treatment affected the change in the SLEB parameter (mm) measured before and after treatment initiation, a mixed model of analysis of variance in the (2) × 2 regimen was calculated. The intra-object factor in the model was the measurement of SLEB taken twice: before treatment and after treatment, while the inter-object factor in the model was the type of treatment administered (systemic vs. topical). Another model of analysis of variance in the (2) × 2 scheme was calculated to check whether age (up to 66 years vs. 66 and above) is a significant factor influencing the change in the SLEB parameter (before treatment vs. after treatment. Another model of the analysis of variance introduced gender as an inter-object factor. In this way, it was checked whether gender could affect SLEB values (before treatment vs. after treatment). 

## 3. Results

**Figure 1:**
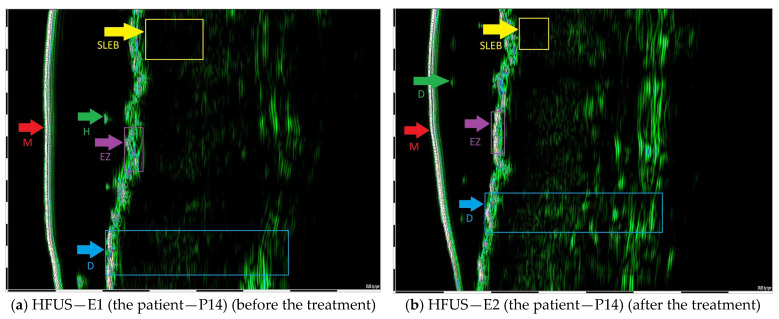
(**a**) The abbreviations from the top: SLEB—subepidermal low echogenic band (1.28 mm); H—hair; M—membrane; EZ—entry zone; D—dermis. (**b**) SLEB (0.78 mm); H—hair; EZ—entry zone; M—membrane; D—dermis. SLEB E2 − SLEB E1 = 0.50 mm.

**Figure 2:**
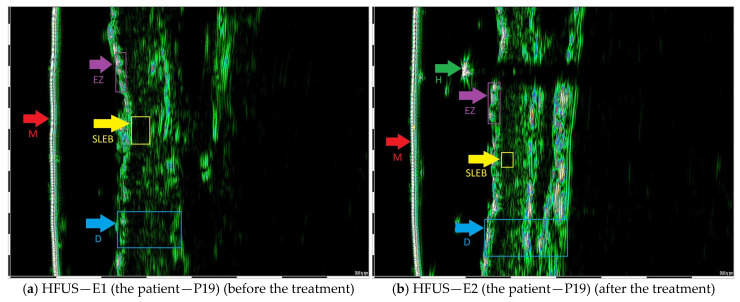
(**a**) The abbreviations from the top: EZ—entry zone; M—membrane SLEB— subepidermal low echogenic band (0.47 mm); D—dermis; (**b**) H—hair; EZ—entry zone; M—membrane SLEB—subepidermal low echogenic band (0.3 mm); D—dermis; SLEB E2 − SLEB E1 = 0.17 mm.

The study involved 31 individuals aged 26 to 88 years (*M* = 62.97; *SD* = 15.83), with the vast majority of the sample being men (*n* = 27; 87.1%), while women constituted only 12.9% (*n* = 4). All the patients had European descent. An age cutoff of 66 years was applied, resulting in 16 patients below 66 years and 15 patients aged 66 years or older (Table 2). At the time of the study, the majority of patients received systemic treatment (*n* = 22; 71%), while 9 participants (29%) underwent topical treatment (Table 2 and Table 3). The duration of the disease ranged from 3 months to 34 years, with a mean duration of 6.59 years (SD = 8.64). In each case, a predefined target lesion was defined and monitored with the HFUS. The first HFUS examination (E1) was performed on the day preceding the initiation of either a new or first-line treatment. The second HFUS assessment (E2) was conducted after 6 weeks of therapy (±2 weeks) average 35 days (Table 3). The primary variable was the difference between initial and early follow-up HFUS measurements (E1–E2), in millimeters. Representative HFUS images illustrating these findings are shown in Figure 1a,b and Figure 2a,b.

The analyses showed the occurrence of a significant SLEB main effect, *F*(1, 29) = 8.88; *p* = 0.006; *η*^2^ = 0.23. This means that there was a significant change between measurements—the value of the SLEB parameter after treatment (*n* = 31, *M* = 0.69, *SD* = 0.89) was lower than its level before treatment (*n* = 31, *M* = 0.9, *SD* = 1.1; *p* = 0.006). The main effect of the type of therapy [*F*(1, 29) = 0.58; *p* = 0.452; *η*^2^ = 0.02] and the effect of the interaction of both factors [*F*(1,29) = 1.18; *p* = 0.286; *η*^2^ = 0.03] were insignificant. This means that on the basis of the study, it cannot be concluded that the type of therapy used affected the change in the SLEB factor (Table 4).

The main effect of the age factor resulted significant: *F*(1, 29) = 4.66, *p* = 0.039, *η*^2^ = 0.14. The effect was that the patients aged up to 66 years (*n* = 32; *M* = 0.44; *SD* = 0.26) had a considerably lower overall SLEB parameter than those aged 66 years and above (*n* = 30; *M* = 1.17; *SD* = 1.32; *p* = 0.039). More importantly, there was also a significant effect of the interaction of the age factor vs. SLEB: *F*(1, 29) = 4.79, *p* = 0.037, *η*^2^ = 0.1. Simple effects tests, taking into account the Bonferroni correction, comparing individual pairs of means, showed that the patients aged 66 years before treatment (*n* = 16; *M* = 0.5; *SD* = 0.26) exhibited lower values of the SLEB parameter than those aged 66 and above (*n* = 15; *M* = 1.34; *SD* = 1.46; *p* = 0.03). A similar effect was observed after treatment (up to 66 years: *n* = 16, *M* = 0.39, *SD* = 0.25 vs. 66 and above: *n* = 15; *M* = 1; *SD* = 1.2), with this effect being significant at the trend level (*p* = 0.058). In addition, it was observed that in the group of patients aged 66 and above, the value of the SLEB parameter after treatment (*n* = 15; *M* = 1; *SD* = 1.2) was lower in comparison with the SLEB value before treatment (*n* = 15; *M* = 1.34; *SD* = 1.46; *p* < 0.001), while this effect did not occur in patients aged up to 66 years (Table 5).

Apart from the isolated effect of the decrease in SLEB values in the second measurement, as compared with the first measurement, no other significant differences were observed. Gender, ignoring other factors, did not differentiate SLEB values [*F*(1, 29) = 0.79; *p* = 0.383; *η*^2^ = 0.03], nor did it interact with the double SLEB measurement [*F*(1, 29) = 0.48; *p* = 0.492; *η*^2^ = 0.01]. This means that the change in the SLEB parameter value in the group of women and men was similar. However, this result should be treated with caution due to the insufficient number of women in the sample (Table 6).

## 4. Discussion

HFUS has rapidly emerged as an invaluable, non-invasive, and cost-effective imaging modality in dermatology. HFUS provides the high-resolution, real-time visualization of the skin, enabling detailed evaluation of the epidermis, dermis, and subcutaneous compartments—structures that are critically involved in the pathology and progression of MF/SS. Importantly, HFUS allows for quantitative assessment of key parameters such as a skin thickness, lesion volume, echotexture, and vascularity, offering objective metrics to monitor disease progression and therapeutic response [14]. The technique is well-tolerated, repeatable, and free from ionizing radiation, making it ideally suited for longitudinal follow-up in both clinical trials and routine practice.

### 4.1. HFUS vs. mSWAT

Accurate assessment of disease extent remains a cornerstone in the management of MF/SS, as staging directly influences therapeutic strategies and provides critical prognostic insight. In this context, precise and reproducible tools are essential for evaluating treatment outcomes and guiding clinical decision-making. Currently, the modified Severity-Weighted Assessment Tool (mSWAT) represents the standard for quantifying cutaneous disease burden in MF/SS and is widely utilized both in clinical practice and in clinical trials. mSWAT enables semi-quantitative estimation of skin involvement by different lesion types, expressed as a percentage of total body surface area (BSA). However, despite its widespread adoption, mSWAT has notable limitations: it remains a largely subjective instrument dependent on clinical judgment, which contributes to inter- and intra-observer variability. Furthermore, its capacity to differentiate among the full morphological spectrum of MF lesions is restricted, potentially impacting the precision of both staging and response evaluation. In our previous study [15], 16 dermatology residents with varying experience levels independently evaluated 14 patients diagnosed with MF/SS using the mSWAT tool and standardized clinical photographs. Their assessments were compared to expert reference scores. Discrepancies occurred in 64.3% of cases, particularly among erythrodermic SS patients. Furthermore, tumors and infiltrative lesions were frequently misclassified, often mistaken for patches or plaques, leading to underestimation of disease severity. The greatest inconsistencies were observed in the evaluation of infiltrative lesions [15]. Our findings are consistent with those of Fernandez-de-Misa et al. [16], who underscored the inherent subjectivity of the mSWAT scoring system. In their study, interrater agreement for patch–plaque assessment reached 86%; however, the Cohen’s κ coefficient was only 0.67, indicating moderate reliability. These results highlight the variability in clinical evaluation and support the need for incorporating objective, standardized methods alongside traditional assessment tools.

Additionally, in 2007, Scarisbrick et al. [17] assessed interobserver variability in mSWAT scoring after standardized training. Despite the session, significant discrepancies persisted, especially in hypopigmented MF, underscoring the subjectivity of assessments influenced by individual experience. While both their study and ours highlight this variability, Scarisbrick focused on BSA estimation rather than lesion type classification. These findings underscore the need for more objective, standardized, and lesion-specific assessment methods to improve staging and outcome evaluation in MF/SS. Although HFUS provides objective and reproducible assessment of skin infiltration depth in MF/SS, it does not capture the extent of surface involvement. Therefore, it should be considered a complementary tool rather than a replacement for clinical scoring systems such as mSWAT. A combined clinical–HFUS approach may offer a more comprehensive and quantitative evaluation, supporting personalized disease monitoring and treatment planning.

### 4.2. HFUS vs. RCM and Dermatoscopy

Our findings should also be considered in the context of other diagnostic approaches that have been investigated for CTCL. Dermoscopy and reflectance confocal microscopy (RCM) have been proposed as useful tools not only for diagnosis but also for assessing disease severity and monitoring remission. Melhoranse Gouveia et al. [18] analyzed 38 MF lesions by RCM, with 19 lesions re-evaluated after 6 months, and reported that an RCM-based checklist combining four features—Pautrier’s microabscesses, epidermal and junctional lymphocytes, and interface dermatitis—was significantly associated with disease severity. Similarly, Soliman et al. [19] examined 88 MF patients using H&E-stained sections, identifying CD3, CD4, and CD8 positivity with CD7 negativity. In addition, dermoscopy revealed characteristic patterns, including a non-homogeneous pink to erythematous background, orange discoloration, whitish scales, dotted and short linear vessels, and spermatozoa-like vessels, with features varying across MF subtypes. Together, these findings highlight the potential role of dermoscopy and RCM as complementary methods in the diagnostic and monitoring armamentarium for CTCL.

### 4.3. HFUS in MF/SS

There are several studies [3,20,21,22,23,24,25] that have systematically examined modifications in the superficial layer of the epidermal barrier (SLEB) and elucidated its potential as a non-invasive biomarker for assessing disease dynamics and treatment responsiveness in patients diagnosed with MF/SS. These investigations underscore the relevance of SLEB thickness and structural alterations as surrogate indicators of disease burden, progression, and therapeutic efficacy.

The study by Polańska et al. [20] was among the first to investigate the utility of HFUS in evaluating therapeutic response in MF. In a cohort of 18 patients with confirmed MF changes in SLEB thickness and total skin thickness were assessed before and after 7–10 weeks of phototherapy, comprising either UVA1 (*n* = 6) or PUVA (*n* = 12). The SLEB was detected in all clinically lesional skin, while it was absent in uninvolved areas, regardless of disease stage. When comparing SLEB values across the two analyses, both studies demonstrated a significant reduction following phototherapy. In Polańska et al. [3] the mean SLEB thickness decreased from 0.256 mm before treatment to 0.064 mm after treatment, with the reduction reaching high statistical significance (*p* < 0.001) and complete disappearance observed in 66% of patients. In contrast, in our study the baseline values were higher (0.50 mm) and post-treatment values also remained higher (0.36 mm), yet the statistical strength of the effect was even more pronounced. Specifically, the paired-samples *t*-test indicated a large effect size (*d* = 1.09, *p* = 0.044), and the Wilcoxon signed-rank test confirmed the finding with *p* = 0.027 and a perfect matched-pairs rank biserial correlation (*r* = 1.00). Thus, although the absolute reduction in SLEB was smaller in our study, the magnitude and robustness of the statistical effect suggest stronger evidence of treatment efficacy compared to the previous study. Collectively, these findings reinforce the value of HFUS as a non-invasive imaging modality for quantifying dermal infiltration and monitoring treatment response in MF/SS. The observed reduction, and in some cases resolution, of the SLEB may serve as a surrogate marker of disease activity and therapeutic efficacy, particularly in early-stage MF.

In a longitudinal study by Polańska et al. [3], three patients with MF were followed for five years to evaluate treatment-related changes in SLEB thickness using HFUS. In our larger cohort of 31 MF patients, treated with a range of modalities including systemic agents (methotrexate, brentuximab, deflazacort), phototherapy (PUVA, nbUVB 311 nm), and topical therapies (chlormethine gel, clobetasol cream 0.05%), we likewise observed a statistically significant reduction in dermal thickness as assessed by HFUS. When comparing our findings with those of Polańska et al. [3], both studies consistently demonstrated a significant post-treatment decrease in SLEB thickness, although the scale and interpretation of the results differ. In Polańska et al. [3], based on only three patients followed over five years, mean SLEB decreased markedly from 0.44 mm to 0.13 mm (*p *= 0.001), and the complete disappearance of SLEB was associated with complete remission. While these observations underline the potential of HF-USG as a long-term monitoring tool, the very small sample size limits the generalizability of the conclusions. In our study, which included a larger cohort (*N* = 31), we also observed a significant reduction in SLEB from 0.90 mm to 0.69 mm (*p *= 0.006, *η*^2^ = 0.23). Importantly, age emerged as a relevant factor: patients ≥ 66 years had higher baseline SLEB values and showed clearer reductions after treatment, whereas younger patients did not. Although the absolute decrease in SLEB was smaller than in Polańska’s report, the broader sample in our study provides additional insight by highlighting demographic influences on treatment response. Residual SLEB was detectable in most patients post-treatment, but its consistent reduction across therapies supports its value as a quantitative marker in MF. In line with Polańska et al. [3], our findings indicate that complete SLEB resolution may accompany full remission, whereas partial yet significant reductions can still represent meaningful therapeutic response. Persistence of SLEB may therefore signal residual disease and justify closer follow-up or treatment adjustment.

In another study, Polańska et al. [21], investigated the relationship between ultrasonographic findings and histological features in a cohort of 10 patients diagnosed with MF. The analysis revealed a mean SLEB thickness of 0.488 mm, with significantly greater values observed in lesions at the plaque stage (0.544 mm) compared to those at the patch stage (0.265 mm). These findings are correct for our study. Importantly, a strong positive correlation was identified between SLEB thickness and the extent of lymphocytic infiltration on histological examination (*r* = 0.994, *p* < 0.01), indicating that SLEB may serve as a reliable non-invasive surrogate marker for dermal lymphocytic burden in MF.

In the study by Wang et al. [22] involving 26 patients with confirmed MF/SS, distinct imaging patterns were observed across disease stages and subtypes. Among the classical MF cases, 16 patients with early-stage lesions consistently demonstrated a SLEB, with only minimal extension into the superficial dermis noted in three plaque-stage lesions. In contrast, seven patients with tumor-stage MF exhibited significantly deeper infiltration, reaching the deep dermis or subcutaneous fat. Quantitative analysis revealed statistically significant differences between early and advanced stages in terms of infiltration depth (*p* < 0.001), Additionally, unique ultrasonographic features were identified in two cases of folliculotropic MF and one case of SS, characterized by a sharply demarcated SLEB and focal hypoechoic areas clustered around dermal hair follicles. These findings highlight the diagnostic value of ultrasound in staging and subclassifying MF/SS based on specific structural and echogenic criteria.

In a case report by Janowska et al. [23], ultra-high-frequency ultrasound (UHFUS, 70 MHz) was used to evaluate the therapeutic response to chlormethine gel in a 63-year-old patient with early-stage MF resistant to clobetasol cream and phototherapy. After two months of treatment with chlormethine gel applied three times weekly, UHFUS demonstrated marked clinical improvement, including resolution of a previously identified 0.94 mm SLEB, vascular lacunae, and periadnexal infiltration. These imaging changes corresponded with complete clinical resolution, supporting both the efficacy of chlormethine gel in refractory lesions and the diagnostic potential of UHFUS. In our cohort, among patients treated with chlormethine gel, HFUS analysis revealed a statistically significant reduction in mean SLEB thickness from 1.244 mm to 1.033 mm (mean reduction: 0.211 mm, *p* = 0.00391). Although the imaging modality used in our study employed conventional HFUS (18–22 MHz), rather than UHFUS devices, the direction and significance of change align with the findings reported by Janowska et al. [23]. These results further reinforce the clinical and imaging-based efficacy of chlormethine gel in treating MF, particularly in early or treatment-resistant disease. Moreover, they demonstrate that HFUS—even at conventional frequencies—is sensitive enough to detect meaningful structural improvement in the dermis following topical therapy.

Although HFUS provides valuable adjunctive information, its diagnostic limitations necessitate that it should not replace histological examination in cases where CTCL is suspected. Mandava et al. [24] undertook a retrospective multicenter analysis to delineate the sonographic characteristics of cutaneous B-cell lymphoma (CBCL) and CTCL. Their evaluation revealed that CTCL plaques commonly exhibited irregular, hypoechoic dermal infiltrates on ultrasound imaging. Nevertheless, the authors concluded that such findings were not unique to CTCL and lacked sufficient specificity to serve as definitive diagnostic markers.

This observation is corroborated by the study conducted by Wohlmuth-Wieser et al. [25], which systematically assessed the utility of HFUS in distinguishing CTCL from other chronic inflammatory dermatoses. Their results demonstrated that sonographic parameters—specifically the regularity of the epidermal surface and the presence or absence of internal echoes within dermal structures—did not reliably differentiate MF patch-stage lesions from AD, nor CTCL plaques from psoriatic lesions. These findings collectively highlight the limited diagnostic resolution of morphological ultrasound features in the context of CTCL, emphasizing the need for adjunctive diagnostic modalities to achieve accurate clinical differentiation.

In contrast to the study by Mandava et al. [24] and Wohlmuth-Wieser et al. [25] recent research by Niu et al. [22] conducted a comparative ultrasonographic analysis to distinguish MF (*n* = 19) from psoriasis vulgaris and eczema (*n* = 48). The study revealed that early MF was characterized by significantly thinner SLEB (*p* = 0.006) relative to the inflammatory dermatoses. Receiver operating characteristic (ROC) analysis identified an SLEB thickness threshold of 0.2655 mm (sensitivity 55.6%, specificity 90.9%) as potential discriminative markers. These findings underscore the value of high-frequency ultrasound as a non-invasive diagnostic tool in distinguishing early-stage mycosis fungoides from clinically similar inflammatory dermatoses, particularly when conducted using advanced imaging systems and interpreted by experienced clinicians. However, it should be noted that histological examination remains the gold standard for the diagnosis of MF/SS. HFUS may serve as a supportive diagnostic tool but cannot replace the definitive assessment by a pathologist.

## 5. Conclusions

This study provides valuable data on the role of HFUS in monitoring treatment response in MF/SS Treatment resulted in a significant reduction in SLEB thickness (0.90 mm → 0.69 mm; *F*(1,29) = 8.88; *p* = 0.006; *η*^2^ = 0.23). The type of therapy, whether systemic (0.75 mm post-treatment) or topical (0.52 mm post-treatment), did not significantly influence this effect (*p* = 0.452). Age proved to be an important factor, as patients ≥ 66 years showed higher baseline SLEB values and a significant reduction after treatment (1.34 → 1.00 mm; *p* < 0.001), whereas younger patients did not exhibit a comparable change. This result provides a basis for further genetic and immunological studies to elucidate the underlying mechanisms. Gender differences were not statistically significant, although women presented lower SLEB values both before and after treatment compared with men. The male predominance in our group is consistent with the epidemiological patterns. Overall, these findings indicate that treatment leads to a measurable reduction in SLEB irrespective of therapy type, with age emerging as a key modifier of response, while gender has no significant impact.

However, several limitations should be acknowledged. The single-center design and relatively small cohort limit the generalizability of the findings. Additionally, the short follow-up period (mean: 35 days) captures only early treatment effects and does not reflect long-term disease dynamics or remission durability. The importance of longitudinal HFUS monitoring with longer-term follow-up should be addressed in future studies. Histological correlation of post-treatment HFUS findings was not systematically performed, which limits insight into the microscopic accuracy of sonographic changes. Although routine histological processing may distort the skin’s anatomy and lead to tissue shrinkage, which is particularly evident in the dermis

Moreover, HFUS remains an operator-dependent technique, and the absence of standardized imaging protocols for MF/SS may reduce reproducibility across clinical settings.

Future multicenter studies with larger, more diverse populations and extended follow-up are needed. Standardization of imaging protocols and integration with histopathologic and clinical endpoints will be essential to fully establish HFUS as a reliable, quantitative tool in the diagnosis and management of MF/SS.

## Figures and Tables

**Table 1:** HFUS features across clinical variants and stages of MF/SS (own experiences and [12,13]).

Selected CTCL Type	HFUS Characteristics
MF—Patch stage (early stage)	The epidermis with sharply demarcated margins and no detectable posterior acoustic enhancement. SLEB demonstrates a homogeneous echotexture throughout.
MF—Plaque stage (early stage)	The epidermis with an irregular, undulating contour and well-defined margins, absence of posterior acoustic enhancement. SLEB is presented with a homogeneous echotexture.
MF—Tumor stage (advance stage)	The epidermis with a wavy and irregular contour and poorly defined margins. Hypoechoic infiltration extends into the deep dermis and subcutaneous tissue.
Folliculotropic MF	SLEB exhibited well-defined boundaries and a homogeneous echotexture. Patchy hypoechoic foci with ill-defined margins are observed surrounding dermal hair follicles. Additionally, round hyperechoic deposits are noted in association with the hair follicles within the dermis.
SS	The epidermis is even and continuous. Patchy hypoechoic foci with indistinct margins are observed in the periadnexal regions surrounding hair follicles.

SLEB—Subepidermal low-echogenic band.

**Table 2:** The characteristics of patients with MF—gender, age and treatments.

		*n*	%
Gender	Male	27	87.1%
	Female	4	12.9%
Age	To 66 years	16	51.6%
	From 66 and above	15	48.4%
Treatment during SLEB	Systemic	22	71.0%
	Topical	9	29.0%

SLEB—subepidermal low-echogenic band.

**Table 3:** Clinical characteristics, treatment modalities, and SLEB Thickness among patients with mycosis fungoides (MF)/Sézary syndrome (SS).

Patient	Diagnosis	Gender	Treatment	E1 [mm]	E2 [mm]	E1–E2 [mm] Difference	Examined Body Region
P1	MF	m	MTX 15 mg p.w. plus clobetasol propionate cream 0.05% b.i.d.	0.42	0.29	0.13	E FR L
P2	MF	m	chlormethine gel q.d.	0.55	0.44	0.11	E FR R
P3	MF	m	chlormethine gel q.d.	0.18	0.11	0.07	F SH R
P4	MF	m	chlormethine gel q.d.	0.67	0.48	0.19	F AR R
P5	MF	m	MTX 25 mg p.w. plus chlormethine gel q.d.	1.02	0.81	0.21	E TH L
P6	MF	f	deflazacort 6 mg q.d. plus chlormethine gel q.d.	0.24	0.14	0.1	F AF R
P7	SS	m	brentuksymab plus deflazacort 6 mg q.d.	0.56	0.43	0.13	F AR R
P8	MF	m	MTX 15 mg p.w.	0.34	0.25	0.09	F AF L
P9	MF	m	chlormethine gel q.d.	0.7	0.64	0.06	F AR L
P10	MF	m	MTX 15 mg p.w.	0.31	0.25	0.06	F AF R
P11	MF	f	nbUVB (311 nm)	0.29	0.26	0.03	F AF R
P12	MF	m	nbUVB (311 nm)	0.35	0.21	0.14	F AF L
P13	MF	m	clobetasol propionate cream 0.05% b.i.d.	0.41	0.27	0.14	F AF R
P14	MF	m	MTX 15 mg p.w.	1.28	0.78	0.5	E TH R
P15	MF	f	clobetasol propionate cream 0.05% b.i.d.	0.4	0.27	0.13	E FR L
P16	MF	m	nbUVB (311 nm)	0.41	0.36	0.05	E FR R
P17	MF	m	MTX 15 mg p.w.	0.56	0.44	0.12	E FR L
P18	MF	m	clobetasol propionate cream 0.05% b.i.d.	0.73	0.65	0.08	E FR R
P19	MF	m	MTX 15 mg p.w.	0.47	0.3	0.17	E FR L
P20	MF	m	PUVA	1.12	0.76	0.36	E TH R
P21	MF	m	MTX 15 mg p.w.	0.59	0.48	0.11	E FR L
P22	MF	m	MTX 20 mg p.w.	0.52	0.45	0.07	E FR L
P23	MF	m	MTX 15 mg p.w.	0.47	0.35	0.12	E FR L
P24	MF	m	MTX 20 mg p.w.	1.01	0.93	0.08	E TH L
P25	SS	m	PUVA	0.33	0.28	0.05	F AF R
P26	MF	m	PUVA, MTX 15 mg p.w.	3.17	1.45	1.72	E TH R
P27	MF	m	MTX 15 mg p.w.	2.6	2.32	0.28	E TH R
P28	MF	m	MTX 15 mg p.w.	0.3	0.17	0.13	F AF L
P29	MF	m	PUVA	0.51	0.31	0.2	E TH R
P30	MF	m	MTX 20 mg p.w.	0.55	0.41	0.14	E AR R
P31	fMF	m	chlormethine gel q.d. and RT	5.71	4.85	0.86	E TR L
P32	MF	m	chlormethine gel q.d.	1.3	1.19	0.11	E TH R
P33	MF	f	chlormethine gel q.d.	0.83	0.64	0.19	E TH L

Abbreviations in alphabetical order: AF—antecubital fossa, AR—arm, E—extension, E1—examination 1, E2—examination 2, F—flexion, fMF—folliculotropic MF, FR—forearm, L—left, MTX—methotrexate, nbUVB (311 nm)—narrowband ultraviolet B 311 nm, PUVA—psoralen plus ultraviolet A, R—right, RT—radiotherapy, SH—shoulder, TH—thigh, TR—trunk.

**Table 4:** SLEB parameter before and after treatment initiation vs. type of treatment (systemic vs. topical) in patients with MF.

		*N*	M	SD	F	*p*	*η* ^2^	Post Hoc
A	before treatment	31	0.90	1.10	8.88	0.006	0.23	B < A **
B	after treatment	31	0.69	0.89				
I	systemic	44	0.88	1.16	0.58	0.452	0.02	n.s.
II	topical	18	0.58	0.31				
A.I	before treatment systemic	22	1.01	1.29	1.18	0.286	0.03	n.s.
A.II	before treatment topical	9	0.64	0.32				
B.I	after treatment systemic	22	0.75	1.04				
B.II	after treatment topical	9	0.52	0.32				

** *p* < 0.01. Abbreviations in alphabetical order: F—ANOVA test statistic, M—mean, *N*—sample size, *p*—*p*-value/statistical significance, SD—standard deviation, *η*^2^—effect size (eta-squared), n.s.—not significant.

**Table 5:** SLEB parameter before and after treatment initiation vs. age in patients with MF.

		*N*	M	SD	F	*p*	*η* ^2^	Post Hoc
A	before treatment	31	0.90	1.10	16.59	0.000	0.33	B < A **
B	after treatment	31	0.69	0.89				
I	to 66 years	32	0.44	0.26	4.66	0.039	0.14	I < II *
II	from 66 and above	30	1.17	1.32				
A.I	before treatment to 66 years	16	0.50	0.26	4.79	0.037	0.10	A.I < A.II *
A.II	before treatment from 66 and above	15	1.34	1.46				B.I < B.II ^†^
B.I	after treatment to 66 years	16	0.39	0.25				B.II < A.II **
B.II	after treatment from 66 and above	15	1.00	1.20				

* *p* < 0.05, ** *p* < 0.01; ^†^ *p* < 0.1. Abbreviations in alphabetical order: F—ANOVA test statistic, M—mean, *N*—sample size, *p*—*p*-value/statistical significance, *η*^2^—effect size (eta-squared).

**Table 6:** SLEB parameter before and after treatment initiation vs. gender in patients with MF.

		*N*	M	SD	F	*p*	*η* ^2^	Post Hoc
A	before treatment	31	0.90	1.10	3.97	0.056	0.12	B < A
B	after treatment	31	0.69	0.89				
I	Male	54	0.86	1.06	0.79	0.383	0.03	n.s.
II	Female	8	0.38	0.23				
A.I	before treatment male	27	0.97	1.16	0.48	0.492	0.01	n.s.
A.II	before treatment female	4	0.44	0.27				
B.I	after treatment male	27	0.74	0.95				
B.II	after treatment female	4	0.33	0.22				

Abbreviations in alphabetical order: F—ANOVA test statistic, M—mean, *N*—sample size, n.s.—not significant, *p*—*p*-value/statistical significance, SD—standard deviation, *η*^2^—effect size (eta-squared).

## Data Availability

The data supporting the findings of this study are not publicly available due to ethical, legal, and privacy restrictions.
